# Beriberi (Thiamine Deficiency) and High Infant Mortality in Northern Laos

**DOI:** 10.1371/journal.pntd.0003581

**Published:** 2015-03-17

**Authors:** Hubert Barennes, Khouanheuan Sengkhamyong, Jean Pascal René, Maniphet Phimmasane

**Affiliations:** 1 Agence Nationale de Recherche sur le VIH et les Hépatites, Phnom Penh, Cambodia; 2 Institut de la Francophonie pour la Médecine Tropicale, Vientiane, Lao, PDR; 3 Epidemiology Unit, Pasteur Institute, Phnom Penh, Cambodia; 4 ISPED, Centre INSERM U897-Epidemiologie-Biostatistique, Univ. Bordeaux, Bordeaux, France; Emory TravelWell Clinic, UNITED STATES

## Abstract

**Background:**

Infantile beriberi (thiamine deficiency) occurs mainly in infants breastfed by mothers with inadequate intake of thiamine, typically among vulnerable populations. We describe possible and probable cases of infantile thiamine deficiency in northern Laos.

**Methodology/Principal Findings:**

Three surveys were conducted in Luang Namtha Province. First, we performed a retrospective survey of all infants with a diagnosis of thiamine deficiency admitted to the 5 hospitals in the province (2007–2009). Second, we prospectively recorded all infants with cardiac failure at Luang Namtha Hospital. Third, we further investigated all mothers with infants (1–6 months) living in 22 villages of the thiamine deficiency patients’ origin. We performed a cross-sectional survey of all mothers and infants using a pre-tested questionnaire, physical examination and squat test. Infant mortality was estimated by verbal autopsy. From March to June 2010, four suspected infants with thiamine deficiency were admitted to Luang Namtha Provincial hospital. All recovered after parenteral thiamine injection. Between 2007 and 2009, 54 infants with possible/probable thiamine deficiency were diagnosed with acute severe cardiac failure, 49 (90.2%) were cured after parenteral thiamine; three died (5.6%). In the 22 villages, of 468 live born infants, 50 (10.6%, 95% CI: 8.0–13.8) died during the first year. A peak of mortality (36 deaths) was reported between 1 and 3 months. Verbal autopsy suggested that 17 deaths (3.6%) were due to suspected infantile thiamine deficiency. Of 127 mothers, 60 (47.2%) reported edema and paresthesia as well as a positive squat test during pregnancy; 125 (98.4%) respected post-partum food avoidance and all ate polished rice. Of 127 infants, 2 (1.6%) had probable thiamine deficiency, and 8 (6.8%) possible thiamine deficiency.

**Conclusion:**

Thiamine deficiency may be a major cause of infant mortality among ethnic groups in northern Laos. Mothers’ and children’s symptoms are compatible with thiamine deficiency. The severity of this nutritional situation requires urgent attention in Laos.

## Author Summary

Infantile thiamine deficiency (beriberi), is rarely seen today after decades of strong public health attention. Infantile beriberi occurs mainly in infants breastfed by mothers with inadequate intake of thiamine. There is evidence of the persistence of infantile thiamine deficiency in Vientiane, the capital of Laos, but insufficient data from outside Vientiane to justify a policy recommendation. We describe possible and probable cases of infantile thiamine deficiency in northern Laos using retrospective and prospective hospital data. In addition we conducted a cross sectional survey in 22 villages where the infants originated. Infantile thiamine deficiency was quite common in retrospective and prospective (hospitals: 54, villages: 17) and cross-sectional surveys (hospital: 4, villages: 10). A second peak of infantile mortality was observed before 6 months and was associated with a high infant mortality in the villages, 106 per 1000 live births (95%CI: 86–128). A total of 60 pregnant mothers and 70 lactating women showed signs of thiamine deficiency. This situation requires urgent attention in Laos.

## Introduction

Thiamine (vitamin B1) acts as an important cofactor in metabolism and energy production. It is required for the biosynthesis of neurotransmitters and the production of substances used in defence against oxidant stress [1]. Thiamine deficiency occurs predominantly in populations, in which the diet consists mainly of very poor sources of thiamine such as milled white cereals, including polished rice (the rich thiamin envelop is removed by polishing) and wheat flour, and where other key sources of thiamine (meat, fish, and vegetables) are infrequently consumed [2]. It is also related to diets that are rich in thiaminase, the natural thiamine-degrading enzyme, which is abundantly present in raw and fermented fish sauce (a common Asian delicacy) certain vegetables and roasted insects consumed primarily in Africa and Asia [3]. Thiamine deficiency can develop within 2–3 months from a deficient intake and can cause illness and death [4].

Clinically apparent thiamine deficiency, also known as Beriberi, has historically been described in vulnerable populations such as refugees, prisoners, during times of war [2,5] and in developed countries with alcohol abuse or parenteral nutrition with insufficient thiamine intake in adults [6,7].

More recently, thiamine deficiency outbreaks were described among young healthy Thai construction workers in Singapore in the 1980s [8,9] and among commercial fishermen in Thailand in 2005 [10,11]. Worldwide, outbreaks of thiamine deficiency have recently been reported in Ivory Cost jails [10,12], in the Gambia [13,14], among African Union troops in Mogadishu, Somalia [15], in Brazil [15–18] and the French island of Mayotte [19]. Adult thiamine deficiency was also recently described in Cambodia, China, India, Thailand, Laos and various countries with unbalanced thiamine/thiaminase diets [8,19–24].

Cardiac failure associated with thiamine deficiency has also been described in Japanese teenagers consuming excessive sweet carbonated soft drinks, instant noodles and polished rice [25].

Thiamine deficiency, is rarely seen today in infants after decades of strong public health attention [26]. It is an acute disease mainly affecting infants that are breastfed by women with deficient thiamine levels [7]. The onset of symptoms is often very rapid and the fatality rate is very high with death often occurring within a few days from the onset of symptoms. Historically, the clinical features have been categorized into three main types; the pure cardiac form or wet thiamine deficiency, the aphonic form, and the neurologic or dry form [27]. The more severe form is called Shoshin beriberi and presents as cardiac failure and lactic acidosis [28]. Beriberi poses difficult diagnostic issues and can be a missed diagnosis, as the dry or wet forms can mimic critical illness or polyneuropathies. In addition, clinical manifestations such as tachypnea, chest indrawing, tachycardia and cardiomegaly can suggest other diagnoses [29–31] [32].

The content of thiamine in breastmilk is related to the mother’s thiamine status [7]. Post partum thiamine deficiency in refugee mothers was associated with high infant mortality in Karen refugees [2,5]. The overall infant mortality rate declined from 183 before the recognition of thiamine deficiency to 78 per 1000 live births afterwards. Thiamine deficiency has also been described in breastfeeding Cambodian and Lao mothers [20,33], and pregnant mothers in China [24]. Recent surveys using whole blood thiamine diphosphate (TDP) revealed that thiamine deficiency was associated with cardiac dysfunction and tachypnea in Cambodian infants [32,34].

In developed countries infantile thiamine deficiency outbreaks have recently been described. For example in Israel an outbreak was due to thiamine deficient soya formula, with a high fatality rate and long term sequelae [35]. In the French island of Mayotte, deficiency was related to inadequate nutrition [28,36]. Infantile thiamine deficiency is periodically reported in intensive care units in babies receiving parenteral nutrition without thiamine or babies with malabsorption receiving prolonged but inadequate vitamin supplements [6,28,37,38].

Infantile thiamine deficiency was described in Laos in the sixties [39]. More recently, cases of young infants with cardiac failure in Mahosot Hospital, Vientiane, suggested the persistence of thiamine deficiency as a cause of infantile mortality [40]. Traditional food avoidance during the post-partum period, nutritional habits, and the high rate of childhood stunting (40%) may all be related to thiamine deficiency [33]. A recent publication revealed that clinically unapparent thiamine deficiency was common among sick infants without overt clinical thiamine deficiency admitted in 2003–2004 [41]. Alarming reports have been received from physicians about the possibility of thiamine deficiency in infants with cardiac failure in northern Laos in recent years [23,41].

However, there is insufficient data from outside Vientiane to provide evidence for discussions about thiamine supplementation in the Lao national nutrition strategy. To help fill this gap, we describe possible and probable cases of infant and maternal thiamine deficiency in Luang Namtha province.

## Methods

### Study site

Luang Namtha province is located in the northwest of the country, bordering Myanmar and China. It is one of the country’s poorest areas with a population of 148,797 people, many of whom live in remote mountain villages. Approximately 23.4% of women had at least one ante natal care visit during their most recent pregnancy in the province [32,42]. The infant mortality in the province (112 per 1000 live births) is one of the highest in the country (national rate 70 per 1000 live births) according to the 2005 national census [43]. At the time of this study (2008) the national infant mortality rate was estimated at 48 per 1000 births [44]. The provincial hospital in Namtha district is the referral centre for all 5 districts and the military hospital. It is a 50-bed hospital with 98 medical and non-medical staff in 2007. Approximately 1,400 outpatients are seen each month and 330 are admitted. It was the only health facility in the province with X-ray and surgery. The 4 other district hospitals are Long, Sing, ViengPhoukha and Nalea.

### Study design

**Fig 1:**
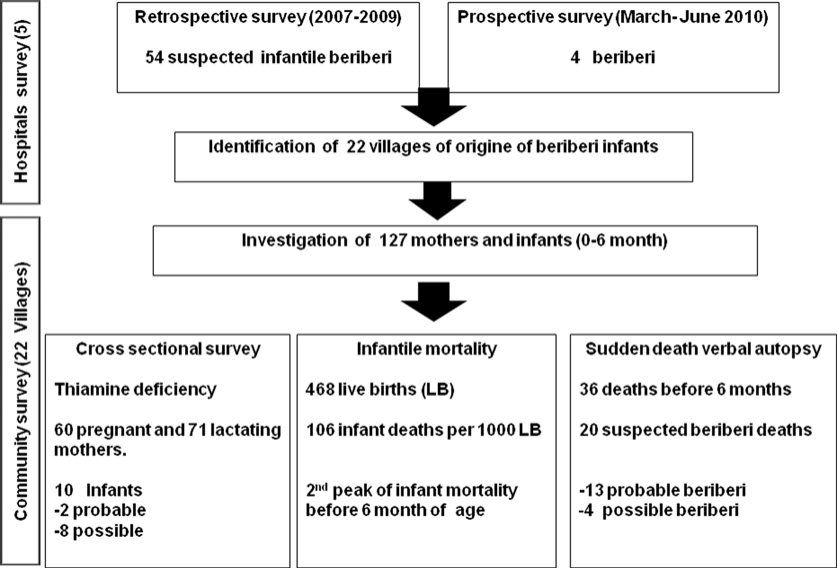
Flow chart of the thiamine deficiency survey in northern Laos.

We conducted various surveys in Luang Namtha province as shown in the flow chart (Fig. 1). First, in a retrospective survey we recorded all infant inpatients with recorded thiamine deficiency in the 5 hospitals in the province between 2007 and 2009. Second, in a prospective survey we recorded all infants admitted with cardiac failure at the emergency ward of Luang Namtha hospital from March to June 2010. Third, from these two surveys we identified 22 villages from where the patients originated and then investigated all mothers with infants (1–6 months) living in these villages. We conducted i) a verbal autopsy of all infants’ deaths and estimated infantile mortality; ii) a cross sectional survey of all mothers and infants (1–6 months) using a pre-tested questionnaire, physical examination and squat test (defined by “the inability of the individual to rise from a squatting position, due to weakness then flaccid paralysis of the lower limbs, without assistance”) [7,12].

### Investigation tools

Characteristics of infants with a discharge diagnosis of thiamine deficiency admitted at the hospitals were retrieved from hospital records. A standardized form was used that included age of infant, main symptoms, treatment received, and response to treatment. An 80 item questionnaire was used in the villages. It included general information on the population (8 questions), presence of a rice-mill, type of rice consumed, socio-economic characteristics (32 questions), maternal food avoidance behaviour, food given to the child in the previous 15 days and information on the children, age by day, sex, mode of birth and detailed information regarding the causes of infants’ death (40 questions).

### Definitions

We used definitions of possible or probable thiamine deficiency for mothers and for infants with sudden cardiac failure or death, based on symptoms and response to thiamine treatment [36].

Possible adult thiamine deficiency was defined in a pregnant women or a mother with a child less than 6 months if she presented or had presented during her pregnancy with at least two of the following signs: motor deficits, paresthesia of the limbs (peripheral numbness, tingling or plantar pain), loss of reflexes, signs of heart failure (jugular swing, cardiac gallop rhythm on auscultation, hepatomegaly), associated with a positive squat test (unable to rise after squatting) [2].

Possible thiamine deficiency in infants was defined as acute symptoms in previously healthy breastfeeding infants associated with cardiac failure (tachypnea> 50/min, tachycardia> 170/min, gallop, hepatomegaly> 3 finger's breadth) or loss of voice. Probable thiamine deficiency was defined if symptoms recovered after thiamine treatment.

Death was diagnosed as due to thiamine deficiency for previously healthy breastfed infants with less than two days of illness fulfilling the possible or probable thiamine deficiency definitions above, and as probable if associated with mother’s symptoms of thiamine deficiency. Due to possible misdiagnosis with acute pneumonia (though infection can precipitate thiamine deficiency [40]) children with signs of pneumonia (cough, fever, +- dyspnea) were excluded if no signs of thiamine deficiency were present in the mother.

The final verbal autopsy diagnosis was proposed during a review meeting of all cases by a committee including one pediatrician, one public health advisor, and two physicians. Only consensual diagnoses were retained.

### Treatment of cases

Mothers suspected of thiamine deficiency were treated with vitamin B1 tablets 100mg, twice daily for 20 days and infants with suspected thiamine deficiency were treated with vitamin B1 tablets, 30 mg per day for 20 days. Patients with acute symptomatic thiamine deficiency received an intramuscular or slow intravenous injection of thiamine (100mg IM for mothers and 50mg for infants). Treatment for associated infection, if any suspected, was provided appropriately. Treatment was provided free of charge. Moreover, all families and village populations received information regarding thiamine deficiency prevention.

### Data management and analysis

Data was entered into EpiData freeware. All records were crosschecked with the original data sheets. Analysis was carried out with STATA, Version 8 (Stata Corporation, College Station, TX, USA). Chi-squared, Fisher’s exact tests and Student’s t-test were used to compare categorical variables and continuous data, respectively. 95% confidence intervals were calculated for continuous and categorical data. We considered p < 0.05 as statistically significant. Infant mortality rates were calculated and compared to the national rate in Laos at the time of survey [44].

### Ethics statement

The study was authorized by the Lao health authorities. Information about the study was provided in Lao language and translated into the local ethnic language by one volunteer from each village. All participants gave informed oral consent in the presence of one village witness as the majority could not read. The procedure of the study was granted ethical approval by the Lao Medical Ethics Committee.

## Results

### Hospital survey

Between 2007 and 2009, 54 infants with sudden onset of cardiac failure were admitted to the 5 hospitals of Luang Namtha province. This number increased from 9 to 24 per year. The infant clinical characteristics and treatment evolution are presented in Table 1. Among them, 20 (37%) probably had an associated infection which may have triggered cardiac failure. Of the 54 infants with cardiac failure, 49 (90.7%) were cured after thiamine administration, three died (5.6%) and two had an unknown status (3.7%). Time of cure was not recorded in patients’ files.

**Table 1:** Characteristics of infants with suspected thiamine deficiency admitted to the 5 hospitals 2007–2009.

	Provincial hospital n = 39 (%)	District hospitals n = 15 (%)	Total n = 54 (%)
Year 2007	9 (23.0)	0	9 (16.6)
Year 2008	6 (15.3)	5 (33.3)	11 (20.3)
Year 2009	24 (61.5)	10 (66.6)	34 (62.9)
Age mean (days)	74 (57.8–95.1)	39 (20.2–77.5)	62 (48.1–80.6)
Female	24 (61.5)	12 (80.0)	18 (33.3)
**Main ethnic group**			
-Hmong	14 (35.9)	2 (13.3)	16 (29.6)
-Khmu	8 (20.5)	4 (26.6)	12 (22.2)
-Aka	3 (7.6)	6 (40.0)	9 (16.6)
-Lenten	4 (10.2)	1 (6.6)	5 (9.2)
-Leu	3 (7.6)	1 (6.6)	4 (7.4)
**Main symptoms**			
-Fever	19 (48.7)	1 (6.6)	20 (37.0)
-Cyanosis	14 (35.8)	1 (6.6)	15 (27.7)
-Cough	12 (30.7)	1 (6.6)	13 (24.0)
-Dyspnea	8 (20.5)	4 (26.6)	12 (22.2)
-Continuous crying	7 (17.9)	5 (33.3)	12 (22.2)
**Evolution under treatment**			
-Cured	37 (94.8)	12 (80.0)	49 (90.7)
-Dead*	2 (5.1)	1 (6.6)	3 (5.5)
-Unknown	0	2 (13.3)	2 (3.7)

*Age at death was: 8 days, 4 and 5 months

Mean and (95% confidence interval)

Four infants with clinical thiamine deficiency were observed during the prospective study at the provincial hospital from March to June 2010. All recovered after thiamine administration and were visited in their own village later. We present a typical description of one patient (Box 1).

**Box 1. Thiamine deficiency in mother and child at provincial hospital (File n° 23).**

Origin: Sonya village, Namtha district.

Mother: 28 years old, Lamet ethnic group, farmer, illiterate, 4^th^ delivery, three children alive.

Pregnancy: pain and paresthesia of upper and lower extremities. The symptoms increased after delivery.

Post partum: strict food avoidance after delivery: first week she ate only glutinous rice and salt, then polished rice, black chicken twice a month.

Physical examination: decreased deep-tendon reflexes; positive squat test.

Infant: aged 41 days.

Symptoms: started by restlessness, irritability and continuous crying, refusal to suckle, dysphonia then aphonia, polypnea, pallor, cyanosis, decreased responsiveness and sleepy; fainting 3–5 times a day. Signs deteriorated during the night. Treated by traditional healer (vacuum or suction cup on the belly).

Physical examination: apyretic, dyspnea, cyanosis, tachycardia, rhonchal fremitus, hepatomegaly with supple belly.

Treatment: Infant treated by IV injection of thiamine 50 mg during 3 days then orally; the mother by tablets.

Evolution: Rapid improvement within hours, they left the hospital after 3 days.

### Village survey

Of 22 villages visited, 18 (81.8%) had an electric rice mill. Of a total of 167 mothers with an infant less than 6 months, 127 mothers and their infants were present and gave consent to be interviewed and undergo a physical examination (Table 2). All mothers consumed polished white rice, 36 (28.3%) had at least one antenatal visit and 28 (22%) reported they received some information on nutrition from health staff during antenatal care. Less than half of the children had received some immunizations (60, 47.2%) (Table 3). Nearly all mothers (125, 98.4%) respected food avoidance after delivery with a median of 30 days.

**Table 2:** Socio-demographic characteristics of mothers in 22 villages of North Laos.

Mother	n = 127 (%)
Median age (years)	23.3 (22.4–24.3)
**Age group**	
16–25 years	87 (68.5)
26–35 years	32 (25.1)
36–45 years	8 (6.2)
**Religion**	
Animist	120 (94.4)
**Education**	
Illiterate	76 (59.8)
Primary	40 (31.4)
Secondary	9 (7.0)
High-school	2 (1.5)
**Profession**	
Farmer	120 (94.4)
Monthly income (US dollars)	148.7 (122.0–181.3)
**Practice of food avoidance**	
yes	125 (98.4)
**Live children**	468
Neonatal deaths	10 (2.1)
Infantile deaths	50 (10.6)

Numbers and (percentages). Median and interquartile. Mean and (95% CI interval).

1 US dollars ~ 8000 kip

**Table 3:** Characteristics of examined infants (0–6 months) in 22 villages of North Laos.

	**Infants n = 127**
Median age (days)	91 (58–139)
Female	55 (43.3)
Place of birth	
Home	94 (74.0)
Hospital*	33 (25.9)
Immunisation	
Yes	60 (47.2)
Thiamine deficiency (TF) suspected	10 (7.8)
TF associated with mother TF	8 (6.2)
Median age (days)	76 (35–106)
Probable TF	2 (1.5)
Possible TF	8 (6.2)
Aphonic form	3 (2.3)
Acute cardiac failure	1 (0.7)
Other signs of wet TF	7 (5.5)

Median age and (Interquartile range)

* One (0.8%) in health centre; 14 (11%) in provincial hospital; 18 (14.1%) in district hospitals

A third of the mothers (45, 35.4%) reported to have had at least one of their children die. Of 468 live born infants, 50 (10.6%, 95%CI: 8.0–13.8) infants died during the first year. Based on this survey, the infant mortality rate was 106 per 1000 live births (95% CI: 86–128).

**Fig 2:**
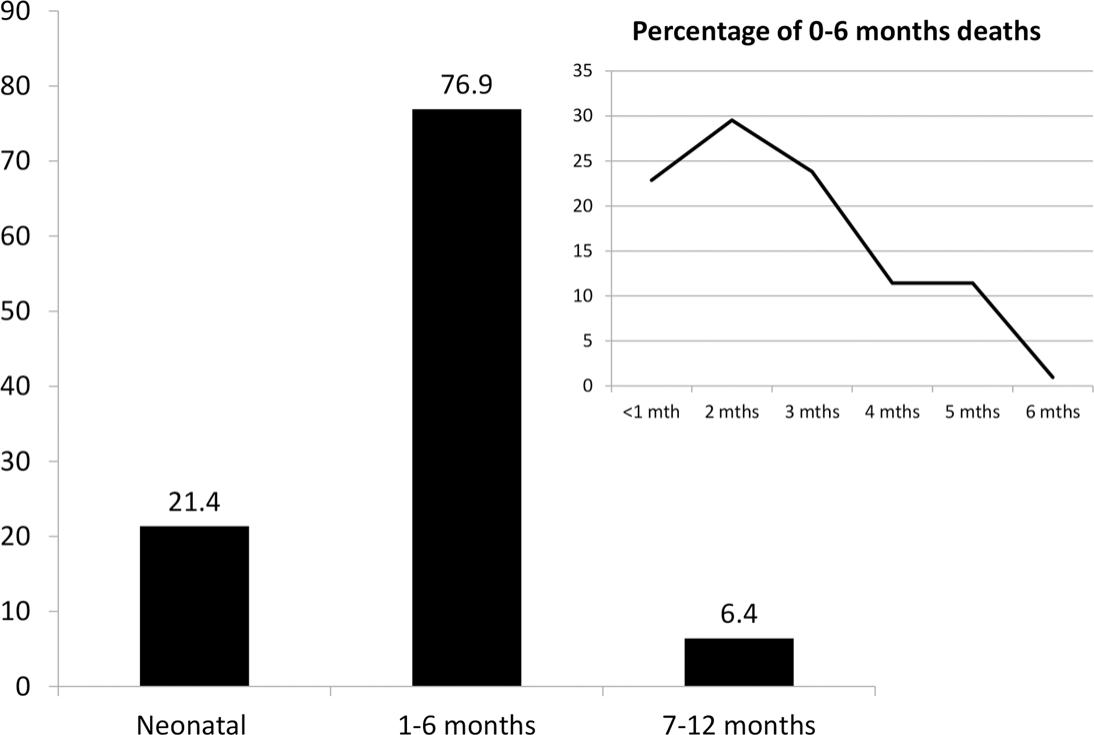
Infantile mortality in 22 villages from northern Laos.

Thirty-six infants (7.6%) died below the age of 6 months. According to mothers, 22.8% of infant deaths occurred during the neonatal period while 29.5% and 23.8% of deaths occurred during the second and third month respectively, and dropped to 11.8% during the fourth and fifth months which suggested a plateau of infantile mortality during the first 3 months of life (Fig. 2). Twenty (10.6%) children presented with sudden death compatible with thiamine deficiency. The verbal autopsy suggested that 17 (3.6%) infants died of thiamine deficiency, 13 (2.7%) as probable and 4 (0.8%) as possible thiamine deficiency. Loss of voice was reported in 10/17 (58.8%). A typical patient is presented in Box 2. For the remaining deaths the verbal autopsy suggested other pathologies (meningitis, laryngitis, convulsions, neonatal infection) as probable causes.

**Box 2. An example of infant thiamine deficiency during the village survey (verbal autopsy) (File n° 24).**

Origin: village of Sonya, Namtha district.

Mother: 45 years old, Lenten ethnic group, farmer, illiterate, 8 children including 3 deaths.

Post partum: strict food avoidance after delivery: only polished rice and salt during one month.

Symptoms: dyspnea, paresthesias and leg pain.

Infant: sudden death at 30 days of age after 24 hours of crying, then dysphonia and unable to suck, dyspnea then cyanosis. He had liquid stools but no fever.

Of 127 mothers, 75 (59.0%) reported edema associated with paresthesia during pregnancy. A total of 60 (47.2%) reported both symptoms and a positive squat test. During the physical examination, 81 (63.7%) complained of paresthesia and 72 (56.6%) of pain in the legs, 71 (55.1%) had a positive squat test, 58 (45.6%) persistent edema and 20 (15.7%) had associated areflexia of upper and lower extremities.

The outcome on mother's symptoms after thiamine oral administration could not be evaluated because of short visits to the villages. One mother could only be considered as probable since she recovered before the team left the village. Finally, possible thiamine deficiency was suspected among 60 women during pregnancy and 71 lactating mothers.

Among 127 infants examined, one had cardiac failure compatible with a probable thiamine deficiency. A total of 10 infants were suspected of thiamine deficiency, two probable, and 8 possible. We present a description (Box 3).

**Box 3. Examples of mother and infant thiamine deficiency observed during the village survey (File n° 25).**

Origin: Sonya village, Namtha district.

Mother: 33 years old, Khamu ethnicity living in a Hmong family, farmer, illiterate. 7 children including 3 deaths at one month, one month and 2 months, all suspected of beriberi (sudden death within 24 hours after refusal to suck, continuous or ceaseless cries, then aphonia, cyanosis, discontinuation of urine and stools).

Post partum: strict food avoidance after delivery: polished glutinous rice only with eggs and chicken 3 times per week.

Symptoms: pain in the legs, paresthesia and walking weakness aggravated by the onset of dyspnea.

Physical examination: abolition of the deep tendon reflex of knees and ankles, positive squat test, dyspnea.

Infant: 65 days old.

Symptoms began with restlessness, irritability and continuous crying, refusal to suckle, vomiting, cries then dysphonia, no fever.

Physical examination: pallor, tachycardia, hepatomegaly.

Treatment: IV injection of thiamine, 100 mg for the mother and 50 mg for the infant

Evolution: Rapid improvement of symptoms after treatment.

File n°117. Origin: Denkang village, Long district.

Mother: 28 years old, Hmong ethnic group, farmer, illiterate, 7 children, 5 deaths.

Post partum: strict food avoidance after delivery: she ate only polished rice, and salt during one month.

Symptoms: edema of legs and arms; paresthesias, dyspnea.

Infant: In the age of 1 month and in good health, he died suddenly after a day with silent screams, cyanosis of the body, and inability to breastfeed. The child was anuric, no liquid stools, no fever, no cough.

File N°153. Origin: Nam-O village, ViengPhouka district

Mother: 25 years old, Khmu ethnic group, farmer, primary school, 2 living children and one child deceased at one month of probable meningitis.

Post partum: food avoidance after delivery: she ate white rice, chicken, rarely vegetable, fish, birds, during 30 days

No clinical symptoms.

Infant: 2 months old. Presence of restlessness, refusal to suck, hoarseness, aphonia, and cyanosis. Symptoms appeared suddenly and severely.

Physical examination: dyspnea, cyanosis, tachycardia and hepatomegaly.

Treatment: The infant was treated by intramuscular injection of thiamine; thiamine tablets given to the mother.

Evolution: after 1 hour the child was able to suckle and no more cry.

## Discussion

This survey suggests the presence of infantile thiamine deficiency and thiamine deficiency in pregnant women and breastfeeding mothers from diverse ethnic groups in Luang Namtha province. Thiamine deficiency was either diagnosed or suspected alone or in association with an infection at the hospital level and in the community. A dramatic therapeutic response to thiamine supplementation was observed in nearly all hospital cases (90.7%). The survey also shows an excessive infant mortality in the 22 villages compared to the national rate [45]. Thiamine deficiency remains a poorly recognized but readily treatable cause of infant death, which strongly advocates for public health prevention and education. We previously recommended using antenatal visits to provide such prevention in Laos [33]. However the rate of antenatal care which varies widely between rural (29%) and urban areas (71%) may limit this recommendation [46]. Extensive public education needs to be conducted with a particular emphasis on mothers belonging to ethnic groups, having a low dietary diversity and those performing hard physical labor or being farmers; three risk factors that were previously pointed out and that also apply to our study population [40]. This survey of highland Lao complements recent work conducted in Vientiane capital where both thiamine deficiency and clinically unapparent thiamin deficiency were described among a majority of lowland Lao (Lao Loum) [39–41]. Another survey shows that 12% of malaria patients (including 165 children less than 15 years of age) had severe biochemical thiamine deficiency in Savannakhet region, southern Laos, without clinical features of thiamine deficiency [23].

The number of thiamine deficiency inpatients increased over the 3 years of the retrospective survey. Over the last years, electricity and electric rice mills were installed in remote villages (H Barennes, personal observations, 2005 to 2012). Traditional foot operated mills which protect the vitamin content of rice, were abandoned and most of the women reported eating mill polished rice which has lost its vitamin B rich envelops. The general practice (98.4% of mothers) of strict food avoidance (eating mostly milled glutinous rice, soaked for hours in water and tea, rich in thiaminases) during the postpartum period further contributes to thiamine deficiency [7,40]. The practice of postpartum food avoidance is very common in Laos and has been associated with a possible reason for infant micro nutrient deficiency and stunting in a prospective survey in 41 randomly selected villages on the outskirts of Vientiane [33]. Nearly all mothers (96.6%) had insufficient thiamine intake [33]. Other possible reasons for the increased diagnosis of thiamine deficiency at the hospitals during the 3 year survey were the arrival of a trained and dedicated pediatrician at the provincial hospital, and the improvement of hospital services which attracted more patients from ethnic groups living outside of the main city (Hubert Barennes, Leila Srour, Gunther Slesak, personal observations). Nevertheless, the increasing rate of thiamine deficiency cases observed at the Luang Namtha hospital might be attributable to a local outbreak related to economic progress and the appearance of electric mills in rural communities of the region. These villages may thus be more at risk than remote communities still relying on foot pounding mills [39].

Before 1991, fatality of infants with congestive heart failure was high at Mahosot Hospital, Vientiane [40]. When infantile beriberi was recognized and measures were taken, clinical response to intravenous thiamine was obtained within 2 hours (15 minutes up to 2 hours) and the in-hospital death rate attributed to thiamine deficiency declined. Nowadays, physicians in Laos are familiar with thiamine deficiency and often treat infants with congestive cardiac failure systematically with parenteral thiamine. However, unapparent clinical thiamine deficiency is still highly prevalent among sick children attending the Vientiane hospital [41] and up to 50–90 children with suspected thiamine deficiency are seen at Mahosot hospital each year. Altogether, this suggests a failure in prevention of this easily curable deficiency.

Detecting thiamine deficiency cases retrospectively from hospital records can be questioned. Hospital records in resource limited settings are usually restricted to basic information. Indeed, the information on treatment efficacy was usually available but not the exact timing of cure. A rapid improvement is usually recognized after parenteral thiamine administration in the wet form of thiamine deficiency [5,40]. This treatment response is different in the neurological forms of thiamine deficiency which usually occur at an older age and were not addressed in this survey [7,35,47,48].

In 2010, in the 22 villages affected by thiamine deficiency, the infant mortality rate was 106 per 1000 live births (95% CI: 86–128), 2.2 fold the 2008 estimated national rate [44]. After neonatal death, instead of decreasing, the mortality remains stable showing a second peak of mortality. A second infant mortality peak before 6 months of age has historically been proposed as a significant feature of a potential thiamine deficiency health problem in the population [4,5,49]. It is important to highlight that public health intervention could reduce the infant mortality as was evidenced with Karen refugees in Thailand [5].

Mothers living in the 22 villages had combinations of all risk factors described for thiamine deficiency [5,40,41]. They were farmers, predominantly illiterate, had low family income, poor dietary diversity and almost all respected food avoidances during the postpartum period.

Typical symptoms of thiamine deficiency were common among women. Though they appeared to be quite common during pregnancy and after delivery, they should be addressed in the context of poor dietary diversity and for women living in similar situations as the one described in rural areas of Cambodia, Thailand, Laos and Vietnam [2,5,20]. During the outbreak in Mayotte among Comorian breastfeeding women, it was similarly reported that mothers complained of paresthesias of lower extremities, pain and some association with walking difficulty, called “lalavy” which resolved after thiamine administration. Interestingly, Comorian mothers had a post partum 40 days regimen which includes a large quantity of a specific ad hoc rice cooked with a lot of water [36].

### Perspectives

Women in Laos should be educated about the importance of a diverse diet before and after delivery and how to maintain a sufficient thiamine intake. Pregnant and lactating mothers must be encouraged to eat unpolished rice, prepare their rice avoiding loss of micronutrients by avoiding unnecessarily long soaking, avoid fermented fish paste, and betel nut chewing during pregnancy and breastfeeding periods. Culturally acceptable ways need to be identified to limit postpartum food avoidance. These measures might be challenging in these populations; hence daily thiamin supplements which are affordable could be considered.

Health professionals should provide nutritional advices during the precious time of the antenatal visits and should be trained to recognize, prevent and treat early symptoms of thiamine deficiency and to offer thiamine supplements to mothers who are not able to systematically comply with dietary advice. For many years, Xayaboury province (north-western Laos) has included thiamine supplementation in the prenatal care program [50]. The midwife in charge of the program reported that thiamine deficiency cases are very uncommon in the province (Leila Srour, personal communication).

Educational campaigns which are now focusing on implementing exclusive breastfeeding in Laos must include thiamine deficiency prevention and detection, as an important component of these educational campaigns. The story of a mother in Savannakhet province helps to understand the context. Attending the district hospital in 2004 with a severely malnourished infant (6 kg at 1 year) she was asked why she fed the infant with coffee creamer. She reported that her first four infants had died before 6 months of age. Suspecting her breastmilk being the problem she decided to feed her fifth child with coffee creamer, as she could not afford expensive infant formula (Leila Srour, personal communication).

Further research is needed to evaluate which preventive strategies are the most effective to reach mothers living in remote villages. Determination of thiamine concentration in breastmilk and the infants’ thiamine status are still needed [40]. Further research is also needed to assess and prevent the hidden consequences of infant thiamine deficiency, especially neurological development and epilepsy [51,52].

A concomitant concern is the need for more accessible and inexpensive tests to evaluate thiamine deficiency, as the current basal erythrocyte transketolase activity (ETK) assays remains unavailable in settings where they are most needed [39]. Another concern is to clarify which tests are most useful. Recently the less conventional whole blood thiamine diphosphate (TDP) concentrations have been used in the field to assess thiamine deficiency [20,34].

### Limitations of the study

This survey has several limitations, including the use of retrospective hospital data and the confounding factor of associated antibiotics together with thiamine administration, the possible recall bias while interviewing mothers regarding the subjective nature of signs such as paresthesia, the lack of laboratory testing for thiamine deficiency, and the use of clinical criteria only to assess thiamine deficiency [2]. Due to time and budget constraints the team could not follow treatment efficacy in the villages and could not validate cases.

We adapted our definitions of thiamine deficiency cases from 2 surveys: one hospital-based survey and one epidemiological outbreak survey [36,40]. Our hospital case definition did not exclude the presence of fever or suspected sepsis since there is evidence that these conditions contribute to precipitating thiamine deficiency [7,41,47]. This may have overestimated the thiamine deficiency frequency but the response to thiamine treatment, an important criterion for diagnosis, was positive in all but three. Conversely, retrospective case review did not include the presence of fever or signs of sepsis and we may have underestimated the number of true thiamine deficiency cases in these high risk populations.

Finally, we screened 22 villages with suspected thiamine deficiency cases but this strategy cannot provide a representative overview of the situation of thiamine deficiency in the region.

### Conclusion

This survey suggests that thiamine deficiency is a major cause of high infant mortality among ethnic groups in northern Laos. Prevention of thiamine deficiency and nutritional education should be addressed on a larger population scale, particularly for pregnant and breastfeeding women, their offspring and their families. It should also focus on at risk Asian populations reporting similar low diversity diets, low thiamine intake, thiaminase rich diets and food avoidance during and after pregnancy.

## Supporting Information

S1 Checklist STROBE checklist. (DOCX)
