# Supplementary material for: Beriberi (Thiamine Deficiency) and High Infant Mortality in Northern Laos
Source: PLoS Negl Trop Dis. 2015 Mar 17;9(3):e0003581. doi: 10.1371/journal.pntd.0003581 (PMC4363666; doi:10.1371/journal.pntd.0003581)
Supplement: S1 Checklist — (DOCX) [file pntd.0003581.s001.docx]

STROBE Statement—checklist of items that should be included in reports of observational studies

|  | Item No. | Recommendation | Page  No. | Relevant text from manuscript |
| --- | --- | --- | --- | --- |
| **Title and abstract** | 1 | (*a*) Indicate the study’s design with a commonly used term in the title or the abstract | *Not included in the title* |  |
|  |  | (*b*) Provide in the abstract an informative and balanced summary of what was done and what was found | 2 | Done in Methodology and principal findings” |
| Introduction | | | |  |
| Background/rationale | 2 | Explain the scientific background and rationale for the investigation being reported | 7 | “there is insufficient data from outside Vientiane to provide evidence for discussions about thiamine supplementation in the Lao national nutrition strategy” |
| Objectives | 3 | State specific objectives, including any prespecified hypotheses | 7 | “we describe possible and probable cases of infant and maternal thiamine deficiency in Luang Namtha province” |
| Methods | | | |  |
| Study design | 4 | Present key elements of study design early in the paper | 8 | Done in study design” |
| Setting | 5 | Describe the setting, locations, and relevant dates, including periods of recruitment, exposure, follow-up, and data collection | 8, 9 | “Study location”, “investigation tools” |
| Participants | 6 | (*a*) *Cohort study*—Give the eligibility criteria, and the sources and methods of selection of participants. Describe methods of follow-up  *Case-control study*—Give the eligibility criteria, and the sources and methods of case ascertainment and control selection. Give the rationale for the choice of cases and controls  *Cross-sectional study*—Give the eligibility criteria, and the sources and methods of selection of participants | 8/9  N/A  8 | in a prospective survey we recorded all infants admitted with cardiac failure at the emergency ward of Luang Namtha hospital from March to June 2010.  all mothers with infants (1-6 months) living in these villages. We conducted i) a verbal autopsy of all infants’ deaths and estimated infantile mortality; ii) a cross sectional survey of all mothers and infants (1-6 months) using a pre-tested questionnaire, |
|  |  | (*b*) *Cohort study*—For matched studies, give matching criteria and number of exposed and unexposed  *Case-control study*—For matched studies, give matching criteria and the number of controls per case |  |  |
| Variables | 7 | Clearly define all outcomes, exposures, predictors, potential confounders, and effect modifiers. Give diagnostic criteria, if applicable | 10 | “Definitions”  definitions of possible or probable thiamine deficiency for mothers and for infants with sudden cardiac failure or death, based on symptoms and response to thiamine treatment  Infant mortality rates were calculated and compared to the national rate in Laos at the time of survey |
| Data sources/ measurement | 8* | For each variable of interest, give sources of data and details of methods of assessment (measurement). Describe comparability of assessment methods if there is more than one group | *9* | *“Investigation tools”* |
| Bias | 9 | Describe any efforts to address potential sources of bias | - | Due to possible misdiagnosis with acute pneumonia (though infection can precipitate thiamine deficiency [40]) children with signs of pneumonia (cough, fever, +- dyspnea) were excluded if no signs of thiamine deficiency were present in the mother.  The final verbal autopsy diagnosis was proposed during a review meeting of all cases by a committee including one pediatrician, one public health advisor, and two physicians. Only consensual diagnoses were retained. |
| Study size | 10 | Explain how the study size was arrived at | N/A |  |

Continued on next page

| Quantitative variables | 11 | Explain how quantitative variables were handled in the analyses. If applicable, describe which groupings were chosen and why | 9 | “Definitions”  And in data management and analysis section |
| --- | --- | --- | --- | --- |
| Statistical methods | 12 | (*a*) Describe all statistical methods, including those used to control for confounding | 11 | “Data analysis” |
|  |  | (*b*) Describe any methods used to examine subgroups and interactions | - |  |
|  |  | (*c*) Explain how missing data were addressed | - |  |
|  |  | (*d*) *Cohort study*—If applicable, explain how loss to follow-up was addressed  *Case-control study*—If applicable, explain how matching of cases and controls was addressed  *Cross-sectional study*—If applicable, describe analytical methods taking account of sampling strategy | - |  |
|  |  | (*e*) Describe any sensitivity analyses | - |  |
| Results | | | | |
| Participants | 13* | (a) Report numbers of individuals at each stage of study—eg numbers potentially eligible, examined for eligibility, confirmed eligible, included in the study, completing follow-up, and analysed | 12 - 13 | Result section |
|  |  | (b) Give reasons for non-participation at each stage |  |  |
|  |  | (c) Consider use of a flow diagram | 8 | “We conducted various surveys in Luang Namtha province as shown in the flow chart (figure 2)” |
| Descriptive data | 14* | (a) Give characteristics of study participants (eg demographic, clinical, social) and information on exposures and potential confounders | 12 | Table1, table 2 |
|  |  | (b) Indicate number of participants with missing data for each variable of interest |  |  |
|  |  | (c) *Cohort study*—Summarise follow-up time (eg, average and total amount) |  |  |
| Outcome data | 15* | *Cohort study*—Report numbers of outcome events or summary measures over time | *29* | Table 1. |
|  |  | *Case-control study—*Report numbers in each exposure category, or summary measures of exposure |  |  |
|  |  | *Cross-sectional study—*Report numbers of outcome events or summary measures | *12* | *“Results; Village survey”* |
| Main results | 16 | (*a*) Give unadjusted estimates and, if applicable, confounder-adjusted estimates and their precision (eg, 95% confidence interval). Make clear which confounders were adjusted for and why they were included |  |  |
|  |  | (*b*) Report category boundaries when continuous variables were categorized |  |  |
|  |  | (*c*) If relevant, consider translating estimates of relative risk into absolute risk for a meaningful time period |  |  |

Continued on next page

| Other analyses | 17 | Report other analyses done—eg analyses of subgroups and interactions, and sensitivity analyses |  |  |
| --- | --- | --- | --- | --- |
| Discussion | | | | |
| Key results | 18 | Summarise key results with reference to study objectives | 14 | Discusion first paragraph |
| Limitations | 19 | Discuss limitations of the study, taking into account sources of potential bias or imprecision. Discuss both direction and magnitude of any potential bias | 19 | “Limitations of the study” |
| Interpretation | 20 | Give a cautious overall interpretation of results considering objectives, limitations, multiplicity of analyses, results from similar studies, and other relevant evidence | 17 | “Perspectives” |
| Generalisability | 21 | Discuss the generalisability (external validity) of the study results | 20 | “we screened 22 villages with suspected thiamine deficiency cases but this strategy cannot provide a representative overview of the situation of thiamine deficiency in the region.” |
| Other information | |  | | |
| Funding | 22 | Give the source of funding and the role of the funders for the present study and, if applicable, for the original study on which the present article is based | 20 | No specific funding, This work was part of a master’s study by “ Institut Francophone pour la Médecine Tropicale” (IFMT, Vientiane, Laos). |

*Give information separately for cases and controls in case-control studies and, if applicable, for exposed and unexposed groups in cohort and cross-sectional studies.

**Note:** An Explanation and Elaboration article discusses each checklist item and gives methodological background and published examples of transparent reporting. The STROBE checklist is best used in conjunction with this article (freely available on the Web sites of PLoS Medicine at http://www.plosmedicine.org/, Annals of Internal Medicine at http://www.annals.org/, and Epidemiology at http://www.epidem.com/). Information on the STROBE Initiative is available at www.strobe-statement.org.
